# Adverse Muscle Composition Is an Early Feature of Chronic Kidney Disease and Associates With Poor Function and Comorbidities

**DOI:** 10.1016/j.xkme.2025.101164

**Published:** 2025-10-30

**Authors:** Ainhoa Indurain, Markus Karlsson, Anders Fernström, Fredrik Uhlin, Jennifer Linge, Mikael Petersson, Olof Dahlqvist Leinhard, Mårten Segelmark

**Affiliations:** 1 Department of Nephrology and Department of Health, Medicine and Caring Sciences, Linköping University, Linköping, Sweden; 2 Department of Health, Medicine and Caring Sciences, Linköping University, Linköping, Sweden; 3 AMRA Medical AB, Linköping, Sweden; 4 Department of Health Technologies, Tallinn University of Technology, Tallinn, Estonia; 5 Center for Medical Image Science and Visualization, Linköping University, Linköping, Sweden; 6 Department of Clinical Sciences, Lund University, Lund, Sweden; 7 Department of Endocrinology, Nephrology and Rheumatology, Skane University Hospital, Lund, Sweden

**Keywords:** Adverse muscle composition, CKD, functional performance, metabolic comorbidity, MRI

## Abstract

**Rational & Objective:**

Sarcopenia is linked to increased morbidity and mortality in chronic kidney disease (CKD), but its definition and assessment vary considerably across studies. This study evaluated the technical feasibility of magnetic resonance imaging (MRI)–based assessment of adverse muscle composition (AMC)—defined as low muscle volume and high fat infiltration—and its associations with comorbidity, functional performance, and risk for coronary heart disease (CHD) in CKD.

**Study Design:**

This is a cross-sectional and prospective study.

**Settings & Participants:**

Participants included 11 patients from a single-center hemodialysis (HD) cohort and 903 individuals with CKD from the UK Biobank, along with matched controls without CKD. For each participant, a personalized muscle volume z-score (sex-specific and body size–specific) was calculated and combined with muscle fat infiltration for AMC evaluation.

**Predictor(s):**

Adverse muscle composition.

**Outcomes:**

Comorbidity index, functional performance (handgrip strength, walking pace, stair climbing, and falls), and new CHD events.

**Analytical Approach:**

In the HD cohort, Spearman rank correlations and Cox proportional-hazards model were used. In the UK Biobank cohort, linear/logistic regression models and Cox proportional-hazards model were used.

**Results:**

AMC was present in 45% of the HD cohort and associated with higher comorbidity index. In the UK Biobank, AMC prevalence was greater in CKD versus no CKD (32% vs 25%, *P* < 0.001). Participants with AMC had worse functional performance (*P* < 0.001), higher comorbidity index (*P* < 0.001), and 2-fold increased CHD incidence (*P* = 0.01).

**Limitations:**

Blood samples defining CKD were taken 7-9 years before MRI, and self-reported data on walking pace, falls, stair climbing, and type 2 diabetes.

**Conclusions:**

The AMC, assessed by MRI, is a prevalent muscle composition phenotype in CKD and is associated with high prevalence of comorbidity, poor function, and increased risk for CHD.

## Plain-language Summary

Sarcopenia is a common and clinically significant complication in chronic kidney disease (CKD), but its definition and assessment remain variable. This study investigated the use of magnetic resonance imaging to identify adverse muscle composition—defined by low muscle volume and increased fat infiltration within muscle tissue. Using data from a hemodialysis cohort and the UK Biobank, we found that adverse muscle composition is more common in individuals with CKD and is significantly associated with increased comorbidity, poor physical performance, and a 2-fold higher risk of new coronary heart disease events. These findings suggest that magnetic resonance imaging–based muscle assessment may help identify high-risk CKD patients earlier and more accurately.

Patients with chronic kidney disease (CKD) have a degenerative muscle process associated with higher muscle degradation, decreased muscle synthesis, and increased muscle fat infiltration (MFI).^1^^,^^2^ This leads to loss of muscle mass and strength and function, and make them more predisposed to sarcopenia leading to a poor quality of life, increased morbidity, hospitalization, and mortality.^3^^,^^4^ Although sarcopenia is a slow and age-related process, a chronic disease state such as CKD may lead to a more rapid decline in muscle mass and function.^5^

Diagnosing sarcopenia in CKD is a challenge as there is no consensus on which operational diagnostic criteria to use when assessing the disease.^6^ However, the current consensus for evaluating sarcopenia recommends assessing muscle volume as well as muscle function (strength or performance).^5^ Because of the high correlation between muscle volume and body size, the patient’s body size needs to be considered when assessing muscle volume. However, the most appropriate method of indexing muscle volume remains uncertain^7^ and could be a limitation for interpreting muscle volume accurately. A recent study using magnetic resonance imaging (MRI) has demonstrated that proper body size–adjustment is pivotal for identifying sarcopenia using muscle volume.^8^^,^^9^

A rapid and standardized MRI scan protocol allowing for an accurate muscle composition analysis by quantification of muscle volume and MFI has been developed.^10^ This assay provides information of muscle quality, and the results have been reported to correlate with low function, adverse outcomes, and mortality.^5^^,^^11^ The combined observation of low muscle volume and high MFI, ie, adverse muscle composition (AMC), has been linked to poor function, metabolic comorbidities and all-cause mortality in nonalcoholic fatty liver disease (NAFLD) as well as all-cause mortality in the general population.^9^^,^^12^^,^^13^

The purposes of this study were, first, to investigate the technical feasibility of MRI-based assessment of AMC in CKD, and second, to analyze the prevalence of AMC in CKD and its associations with comorbidity—assessed using a comorbidity score and the presence of type 2 diabetes (T2D) and chronic heart disease (CHD)—as well as with physical performance, measured using handgrip strength and functional performance questionnaires.

## Materials and Methods

### Participants and Clinical Data

This is a cross-sectional and prospective study. We first performed a pilot study to assess muscle composition and its clinical relevance within advanced CKD. Eleven patients undergoing hemodialysis (HD) at the University Hospital of Linköping were enrolled in 2014.^14^ This cohort has previously been used to study an MRI-based biomarker of tissue hydration and demonstrated its sensitivity to fluid changes before and after dialysis.^14^ Comorbidity score was assessed using the new comorbidity dialysis-specific index (nCI).^15^ Electronic health records were used to calculate the nCI and to retrieve 5-year survival data. The regional ethical review board approved the study protocol (2013/475-31), and written informed consent was obtained from all patients.

**Figure 1:**
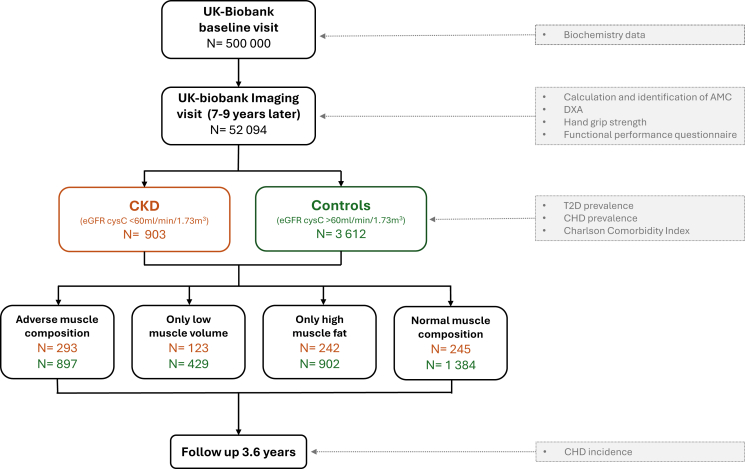
Flow diagram of study data, calculations, and analyses. AMC, adverse muscle composition; CHD, coronary heart disease; DXA, dual-energy x-ray absorptiometry; T2D, type 2 diabetes.

To assess if AMC is prevalent already in earlier stages of CKD, a cohort was generated from the UK Biobank imaging study and compared to a control group. UK Biobank is one of the largest prospective population-based cohorts study following 500,000 volunteers in 2006-2010.^16^ As a sub-study, 100,000 participants are being re-called for a detailed imaging assessment including repeat of baseline assessment. Participants with CKD at the first visit defined as Cystatin C based estimated glomerular filtration rate (eGFR) <60mL/min/1.73m^2^ were included among the 52,094 participants first scanned in the UK Biobank imaging study. The eGFR was calculated according to the Chronic Kidney Disease Epidemiology Collaboration formula based on cystatin C during the UK Biobank baseline visit (∼7-9 years before the MRI-visit).^17^ Participants identified with end-stage kidney disease and kidney replacement therapy (dialysis or transplantation) at time of imaging (detected by using ICD-10 and OPCS4 codes or self-report) were excluded.^18^ Participants with CKD were matched 1:4 with controls (eGFR > 60 mL/min/1.73m^2^) using sex, age, and basic metabolic index (BMI). The Charlson Comorbidity Index (CCI) was used for calculating the comorbidity index score based on the ICD-10 codes from electronic health records.^19^ Diagnosis for CHD or T2D was based on electronic health records in combination with self-reported information collected via interviews with trained nurses. The CHD was defined by ICD-10 codes I20–I25, Z95.1. The T2D data were self-reported but previously diagnosed by a physician and with age at diagnosis above 30 years. Low handgrip strength was defined using the sex-specific cut-offs recommended by European Working Group of Sarcopenia in Older People (EWGSOP2) (<16/27 kg [females/males]).^5^ Data on walking pace, number of falls and stairs climbed were acquired through self-reported touchscreen questionnaires (Item S1). Sarcopenia was defined according to the recommendation by the EWGSOP2 requiring low hand grip strength and low muscle volume (dual-energy x-ray absorptiometry-based appendicular lean mass/height^2^, <5.5/7.0 kg/m^2^ [females/males]).^5^ See also Fig 1 for the study process. The study was approved by the North West Multicenter Research Ethics Committee, United Kingdom. Written informed consent was obtained before study entry (project ID 6569).

### Measurements of Body Composition by MRI

Participants were scanned using rapid whole-body fat and water separated MRI and images were analyzed using AMRA researcher.^8^ For calculations of Muscle_comb_, muscle volume z-score, and sex-adjusted MFI(see definitions below) we used normative data from 40,169 subjects in the UK Biobank (data access application 6,569).^12^

• FFMV: fat-free muscle volume, the viable muscle tissue (volume of all voxels with fat fraction < 50%) in the thighs.^20^^,^^21^
• Muscle volume z-score: For each patient, a virtual control group was stratified among the normative data (minimum 150 individuals) with the same sex and similar BMI. Muscle volume z-score was calculated by measuring how many standard deviations each patient was from the mean thigh FFMV/height^2^ of their virtual control group.^8^ Patients were classified as having low muscle volume if the z-score was below 25th percentile (< −0.68 SD).
• MFI: muscle fat infiltration, the mean fat fraction within the viable muscle tissue of the right and left anterior thighs.^20^^,^^21^ Because of the difference in magnitude between females (higher) and males (lower),^10^^,^^12^ a sex-adjusted MFI was calculated by subtracting the sex-specific population median in the normative data. Patients were classified as having high muscle fat infiltration if MFI was above the sex-specific 75th percentile (for men [7.69%] and women [8.82%] separately).^9^^,^^12^
• Muscle_comb_: Combined muscle score, was estimated by projecting sex-adjusted MFI and muscle volume z-score on the linear regression line describing, in the normative data, the relationship between MFI and muscle volume z-score in the normative data.
• VAT: Visceral adipose tissue volume was defined as the volume of fat inside the abdominal cavity.
• Abdominal subcutaneous adipose tissue was defined as the volume of subcutaneous fat from the top of the femur to the top of the T9 vertebrae.

**Figure 2:**
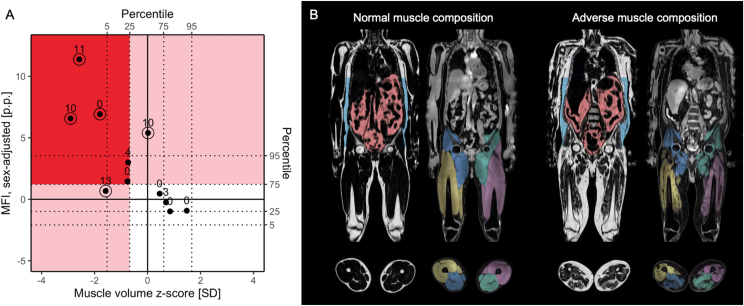
Muscle composition and example images on the HD-cohort. (A) Distribution of muscle volume z-score and sex-adjusted MFI among the patients in the dialysis dataset. The number next to each observation is the nCI score. The 5 observations with circles around correspond to deaths. The dotted lines show the 5th, 25th, 75th, and 95th percentile in the UK Biobank dataset for each variable. The red area corresponds to having adverse muscle composition, the pink areas correspond to having only high MFI or only low muscle volume, and the white area corresponds to having normal muscle composition. (B) Example fat and water images, including segmentations, of patients treated with hemodialysis with normal muscle composition and adverse muscle composition. MFI, muscle fat infiltration; nCI, new comorbidity dialysis-specific index.

Based on the above thresholds, participants were divided into 4 muscle composition phenotypes: (1) normal muscle composition (neither low muscle volume or high MFI), (2) only low muscle volume, (3) only high MFI, and (4) adverse muscle composition (AMC; low muscle volume and high MFI) (Fig 2).

### Statistical Analyses

In the HD cohort, a paired *t* test was used to determine if there was a significant difference between MRI measurements before and after dialysis, and differences between AMC and no-AMC groups were assessed using *t* test. The Spearman rank correlation test was used to analyze the associations between muscle composition variables and comorbidity. To analyze the association between muscle composition variables and mortality, the Cox proportional-hazards model was used.

In the UK Biobank cohort, differences between the CKD and control groups, and between the AMC and no-AMC groups in the CKD-UK Biobank cohort were tested using *t* test or 2-proportion z-test unadjusted and linear/logistic regression models adjusted for age, sex, and BMI were employed. A Cox-proportional-hazards model was used to assess the association between muscle composition phenotypes and CHD incidence. Logistic regression analysis was used for the analysis of the association between muscle composition phenotypes and functional performance, CCI, CHD, and T2D. Statistical analyses were performed using R (version 3.6.2).

## Results

### Muscle Composition Within the HD Cohort

A total of 11 HD patients were enrolled in the pilot study. The majority were men with a mean age of 60.3 ± 12.3 years (Table 1). According to the AMC thresholds,^9^ 6 (54%) patients presented with low muscle volume, 6 (54%) patients with high MFI, and a total of 5 (45%) patients presented with AMC (Fig 2). The muscle volume z-score and MFI did not significantly correlate with comorbidity. However, when combining them into the Muscle_comb_ the correlation was statistically significant (Table 2).

**Table 1:** Characteristics of Patients in Hemodialysis Cohort and Comparison of Hemodialysis Patients With and Without Adverse Muscle Composition

	All HD Patients	HD Without Adverse Muscle Composition	HD With Adverse Muscle Composition	*P*
**N**	11	6	5	-
**Sex (female/male)**	18.2%/81.8%	16.7%/83.3%	20%/80%	-
**Age (y)**	60.3 ± 12.3	55.7 ± 12.5	67 ± 9	0.12
**BMI (kg/m^2^)**	26.7 ± 4.8	27.2 ± 5.4	26.1 ± 4.5	0.72
**Muscle composition**				
FFMV (L)	11.1 ± 2.7	12.5 ± 2.7	9.4 ± 1.5	0.04
Muscle volume z-score (SD)	−0.6 to 1.5	0.3 ± 1.0	−1.8 to 1.0	0.009
MFI (%)	9.8 ± 3.9	7.4 ± 2.3	12.6 ± 3.6	0.03
MFI, sex-adjusted (p.p.)	3.1 ± 4.0	0.7 ± 2.4	5.9 ± 3.9	0.04
Muscle comb	1.6 ± 2.1	0.3 ± 1.2	3.2 ± 1.9	0.02
**Laboratory examinations**				
Hemoglobin (g/L)	117.0 ± 13.1	118.7 ± 6.8	115.2 ± 18.1	0.70
Phosphate (mmol/L)	1.6 ± 0.3	1.5 ± 0.3	1.7 ± 0.3	0.30
Albumin (g/L)	35.8 ± 3.6	36.8 ± 2.6	34.6 ± 4.5	0.37
Ferritin (μg/L)	465.2 ± 275.1	355.5 ± 300.2	596.8 ± 192.2	0.14
**Dialysis characteristics**				
Dialysis duration (mo)	31.4 ± 27.9	46.8 ± 34.9	16.4 ± 12.0	0.09
Dialysis time/week (h)	15.0 ± 3.5	16.3 ± 3.9	13.4 ± 1.5	0.14
AV-fistula/dialysis catheter	81.8%/18.2%	83.3%/16.7%	80%/20%	-
**Causes of end-stage renal diseases**				
Diabetic nephropathy	4 (36.4%)	2 (33.3%)	2 (40%)	-
Chronic glomerulonephritis	3 (27.3%)	2 (33.3%)	1 (20%)	-
Polycystic kidney disease	1 (9%)	0 (0%)	1 (20%)	-
Chronic infravesical obstruction	1 (9%)	1 (16.7%)	0 (0%)	-
Renal cancer/bilateral nephrectomy	1 (9%)	0 (0%)	1 (20%)	-
Unknown	1 (9%)	1 (16.7%)	0 (0%)	-
**Comorbidities**				
Congestive heart failure, n (%)	3 (27%)	2 (33.3%)	1 (20%)	-
Coronary artery disease, n (%)	4 (36%)	2 (33.3%)	2 (40%)	-
Cerebrovascular disease, n (%)	2 (18%)	2 (33.3%)	0 (0%)	-
Diabetes mellitus, n (%)	4 (36%)	2 (33.3%)	2 (40%)	-
Malignancy, n (%)	1 (9%)	0 (0%)	1 (20%)	-

*Note:* For continuous variables, data is reported as mean ± standard deviation.

Abbreviations: FFMV, fat-free muscle volume; HD, hemodialysis; MFI, muscle fat infiltration. Muscle_comb,_ combined measurement; OL-HDF, on-line hemodiafiltration.

**Table 2:** Association Between Muscle Composition Variables and Comorbidity Index (nCI) in Hemodialysis Cohort

	Estimate of Rank Correlation	*P*
**Muscle volume z-score**	−0.5448	0.0831
**MFI, sex-adjusted**	0.4731	0.1416
**Muscle_comb_**	0.6022	0.0499

*Note:* Spearman rank correlation tests.

Abbreviations: MFI, muscle fat infiltration; Muscle_comb._ combined measurement.

**Figure 3:**
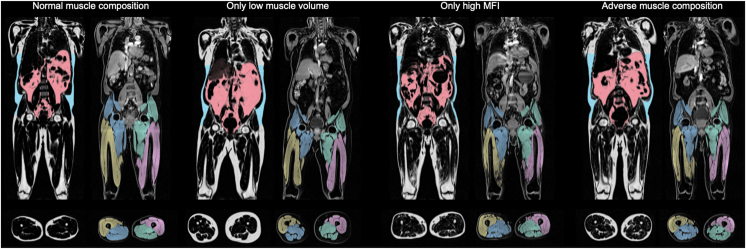
UK Biobank CKD participants with the 4 different muscle composition phenotypes. Example fat and water images, including segmentations. CKD, chronic kidney disease.

During a mean follow-up period of 4.2 years, 5 (45%) of the 11 patients died. Univariate Cox proportional hazard modeling did not show a significant association between AMC (compared with non-AMC) for predicting mortality (HR: 2.07; 95% CI, 0.35-12.44; *P* = 0.426). However, there was a significant association for both muscle volume z-score (HR: 0.33; 95 % CI, 0.13-0.85; *P* = 0.022), sex adjusted MFI (HR: 1.52; 95% CI, 1.07-2.16; *P* = 0.020), and Muscle_comb_ (HR: 2.33; 95% CI, 1.17-4.65; *P* = 0.017). A 2D-plot visualization of muscle volume z-score and sex-adjusted MFI showed that muscle composition was strongly associated with both comorbidity score and death (Fig 3). Data in the dialysis cohort are from MRIs performed before dialysis sessions. However, the MFI did not change before versus after dialysis and the FFMV mean difference was 0.27 ± 0.21.

### Muscle Composition Withing UK Biobank Cohort

After exclusion of 5 participants with kidney replacement therapy, 903 UK biobank participants with CKD were included along with 3,612 controls. Their mean eGFR was 53.5 ± 6.4mL/min/1.73m^2^ and the majority were male (52.2%) (Table 3).

**Table 3:** Characteristics of all Participants in UK Biobank Cohort, the CKD Participants, and the Control Group

	All Participants in UK Biobank Imaging	CKD	Controls	*P*	*P* (Adjusted)
**N**	52,094	903	3,612	-	-
**Sex (female/male), (%)**	51.9%/48.1%	47.8%/52.2%	47.8%/52.2%	-	-
**Age (y)**	65.3 ± 7.7	72.2 ± 5.8	72.2 ± 5.8	0.882	0.864
**Age at visit 1 (y)**	55.4 ± 7.6	62.5 ± 5.5	61.7 ± 5.7	< 0.001	< 0.001
**Height (m)**	169.3 ± 9.1	168.9 ± 9.0	168.6 ± 9.3	0.458	0.254
**Weight (kg)**	76.0 ± 15.1	82.7 ± 17.0	82.4 ± 16.8	0.621	0.222
**BMI (kg/m^2^)**	26.4 ± 4.4	29.0 ± 5.3	29.0 ± 5.3	0.942	0.943
**Kidney function at visit 1**					
Cystatin C (mg/L)	0.9 ± 0.1	1.3 ± 0.2	0.9 ± 0.1	< 0.001	< 0.001
eGFR (mL/min/1.73m^2^)	92.1 ± 14.3	53.5 ± 6.4	84.9 ± 13.0	< 0.001	< 0.001
eGFR > 60 (n [%])	51,186 (98.3%)	0 (0.0%)	3,612 (100.0%)	< 0.001	0.997
eGFR 59-45 (n [%])	817 (1.6%)	814 (90.1%)	0 (0.0%)	< 0.001	0.961
eGFR 44-30 (n [%])	81 (0.2%)	80 (8.9%)	0 (0.0%)	< 0.001	0.968
eGFR 15-29 (n [%])	9 (0.0%)	8 (0.9%)	0 (0.0%)	< 0.001	0.989
eGFR < 15 (n [%])	1 (0.0%)	1 (0.1%)	0 (0.0%)	0.453	0.997
**Laboratory examinations at visit 1**					
Hemoglobin (g/dL)	14.2 ± 1.2	14.3 ± 1.4	14.3 ± 1.1	0.597	0.311
Albumin (g/L)	45.4 ± 2.5	44.5 ± 2.7	44.9 ± 2.5	< 0.001	< 0.001
HbA1c (mmol/mol)	35.0 ± 5.1	37.3 ± 6.5	36.4 ± 5.7	< 0.001	< 0.001
CRP (mg/L)	1.1 (0.6-2.2)	2.2 (1.2-4.7)	1.5 (0.8-2.9)	< 0.001	< 0.001
Testosterone (nmol/L)	6.9 ± 6.1	7.2 ± 5.8	7.1 ± 5.9	0.955	0.525
uACR > 3 mg/mmol	4,352 (8.5%)	125 (14.1%)	272 (7.7%)	< 0.001	< 0.001
**Fat distribution**					
Visceral adipose tissue (L)	3.8 ± 2.3	5.2 ± 2.6	4.9 ± 2.6	< 0.001	< 0.001
Abdominal subcutaneous adipose tissue (L)	6.9 ± 3.2	8.4 ± 3.9	8.2 ± 3.8	0.104	< 0.001
**Muscle composition**					
FFMV (L)	10.1 ± 2.5	10.0 ± 2.3	10.2 ± 2.4	0.021	< 0.001
Muscle volume z-score (SD)	−0.1 ± 1.0	−0.6 ± 1.0	−0.4 ± 0.9	< 0.001	< 0.001
MFI (%)	7.4 ± 1.9	9.1 ± 2.5	8.5 ± 2.2	< 0.001	< 0.001
MFI, sex-adjusted [p.p.]	0.4 ± 1.9	2.1 ± 2.4	1.5 ± 2.1	< 0.001	< 0.001
Adverse muscle composition [n (%)]	6,319 (12.1%)	293 (32.4%)	897 (24.8%)	< 0.001	< 0.001
Only high MFI (n [%])	7,554 (14.5%)	242 (26.8%)	902 (25.0%)	0.277	0.249
Only low muscle volume (n [%])	7,714 (14.8%)	123 (13.6%)	429 (11.9%)	0.169	0.142
Normal muscle composition (n [%])	30,507 (58.6%)	245 (27.1%)	1,384 (38.3%)	< 0.001	< 0.001
**Functional performance**					
EWGSOP2 Sarcopenia (n [%])	501 (1.5%)	18 (3.0%)	33 (1.5%)	0.018	0.018
Low hand grip strength (n [%])	4,764 (9.4%)	158 (18.2%)	514 (14.7%)	0.012	0.011
No stair climbing (n [%])	4,550 (8.8%)	118 (13.5%)	463 (13.0%)	0.777	0.739
Slow walking pace (n [%])	2,644 (5.1%)	161 (18.2%)	433 (12.1%)	< 0.001	< 0.001
>1 fall last year (n [%])	2,594 (5.0%)	66 (7.4%)	205 (5.7%)	0.071	0.061
**Comorbidity**					
Prevalent type 2 diabetes (n [%])	2,719 (5.2%)	130 (14.5%)	313 (8.7%)	< 0.001	< 0.001
Prevalent coronary heart disease (n [%])	2,900 (5.6%)	153 (17.0%)	340 (9.5%)	< 0.001	< 0.001
Charlson comorbidity index	0.4 ± 0.9	1.1 ± 1.6	0.5 ± 1.0	< 0.001	< 0.001

*Note:* For continuous variables, data is reported as mean *±* standard deviation, except non-normally distributed CRP, which is reported as median (inter quartile range). The *P* values represent comparison between participants with chronic kidney disease and the control group tested using 2-proportions z-test (binary variables), *t* test (continuous variables) and logistic/linear regression adjusted for sex, age, and BMI.

EWGSOP2, European Working Group on Sarcopenia in Older People; FFMV, fat free muscle volume; MFI, muscle fat infiltration.

### Differences Between CKD and Control Group

AMC was significantly more prevalent within CKD-UK Biobank participants compared with the control group (32.4% vs 24.8%; *P* < 0.001) (Table 3). On the contrary, only 3.0 % of the CKD group had sarcopenia (EWGSOP2), 2 times more than controls (1.5%) (*P* = 0.02). Within CKD-participants 27.1% had normal muscle composition, 13.6% had only low muscle volume, 26.8% had only high MFI (Table S1).

### Differences Between CKD-Participants With and Without AMC

CKD participants with AMC were older (*P* < 0.001) and presented a lower eGFR (*P* = 0.007) compared with those without AMC (Table 4).

**Table 4:** Comparison of CKD-UK Biobank Participants With/Without Adverse Muscle Composition

	CKD Without Adverse Muscle Composition	CKD With Adverse Muscle Composition	*P*	*P* (Adjusted)
**N**	610	293	-	-
**Sex (female/male)**	49.0%/51.0%	45.4%/54.6%	0.342	1.000
**Age (y)**	71.4 ± 6.0	73.9 ± 5.1	< 0.001	1.000
**Age at visit 1 (y)**	61.9 ± 5.8	63.6 ± 4.6	< 0.001	0.008
**Height (m)**	169.3 ± 9.1	168.0 ± 8.9	0.051	0.002
**Weight (kg)**	82.1 ± 17.4	84.2 ± 16.0	0.074	0.007
**BMI (kg/m^2^)**	28.6 ± 5.4	29.8 ± 4.9	0.001	0.002
**Kidney function at visit 1**				
Cystatin C (mg/L)	1.3 ± 0.2	1.3 ± 0.2	0.150	0.017
eGFR (mL/min/1.73m^2^)	53.9 ± 6.1	52.8 ± 7.0	0.026	0.007
eGFR 45-60 (n [%])	563 (92.3%)	251 (85.7%)	0.003	0.001
eGFR 44-30 (n [%])	42 (6.9%)	38 (13.0%)	0.004	0.002
eGFR 29-15 (n [%])	4 (0.7%)	4 (1.4%)	0.493	0.215
eGFR <15 (n [%])	1 (0.2%)	0 (0.0%)	1.000	0.998
**Laboratory data at visit 1**				
Hemoglobin (g/dL)	14.3 ± 1.4	14.3 ± 1.4	0.466	0.065
Albumin (g/L)	44.6 ± 2.7	44.5 ± 2.8	0.739	0.894
HbA1c (mmol/mol)	36.9 ± 5.3	38.3 ± 8.5	0.013	0.104
CRP (mg/L)	2.1 (1.1-4.3)	2.5 (1.5-4.9)	0.744	0.972
Testosterone (nmol/L)	7.2 ± 6.0	7.0 ± 5.5	0.615	0.054
uACR > 3 mg/mmol (n [%])	82 (13.7%)	43 (14.9%)	0.686	0.175
**Fat distribution**				
Visceral adipose tissue (L)	4.9 ± 2.5	5.9 ± 2.6	< 0.001	< 0.001
Abdominal subcutaneous adipose tissue (L)	8.1 ± 3.9	9.2 ± 3.9	< 0.001	< 0.001
**Muscle composition**				
FFMV (L)	10.4 ± 2.4	9.1 ± 2.0	< 0.001	< 0.001
Muscle volume z-score (SD)	−0.1 ± 0.8	−1.5 ± 0.6	< 0.001	< 0.001
MFI (%)	8.3 ± 2.1	10.8 ± 2.4	< 0.001	< 0.001
MFI, sex-adjusted (p.p)	1.3 ± 2.0	3.8 ± 2.3	< 0.001	< 0.001
Adverse muscle composition (n [%])	0 (0.0%)	293 (100.0%)	< 0.001	0.998
Only high MFI (n [%])	242 (39.7%)	0 (0.0%)	< 0.001	0.971
Only low muscle volume (n [%])	123 (20.2%)	0 (0.0%)	< 0.001	0.976
Normal muscle composition (n [%])	245 (40.2%)	0 (0.0%)	< 0.001	0.975
**Functional performance**				
EWGSOP2 Sarcopenia (n [%])	5 (1.2%)	13 (6.8%)	0.001	< 0.001
Low hand grip strength (n [%])	86 (14.7%)	72 (25.4%)	< 0.001	0.001
No stair climbing (n [%])	69 (11.6%)	49 (17.4%)	0.025	0.090
Slow walking pace (n [%])	84 (14.0%)	77 (27.2%)	< 0.001	0.001
>1 fall last year (n [%])	45 (7.5%)	21 (7.2%)	0.988	0.929
**Comorbidity**				
Prevalent type 2 diabetes (n [%])	71 (11.7%)	59 (20.3%)	0.001	0.049
Prevalent coronary heart disease (n [%])	97 (16.0%)	56 (19.2%)	0.262	0.660
Charlson comorbidity index	0.9 ± 1.4	1.4 ± 1.8	< 0.001	< 0.001

*Note:* For continuous variables, data is reported as mean ± standard deviation, except non-normally distributed CRP, which is reported as median (inter quartile range). The *P* values represent comparison between participants with chronic kidney disease and the control group tested using 2-proportions z-test (binary variables), *t* test (continuous variables), and logistic/linear regression adjusted for sex, age, and BMI.

Abbreviations: EWGSOP2, European Working Group on Sarcopenia in Older People; FFMV, fat free muscle volume; MFI, muscle fat infiltration,.

**Figure 4:**
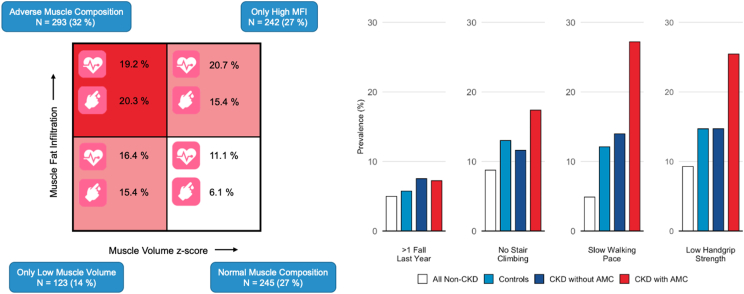
Muscle composition, comorbidity, and functional performance in CDK-UK Biobank cohort. Square muscle composition plot includes prevalence of coronary heart disease and type 2 diabetes. Bar plots show prevalence of poor function. CKD, chronic kidney disease.

**Figure 5:**
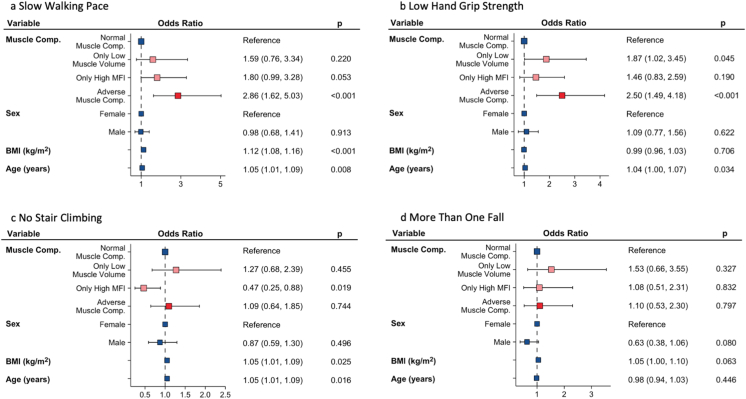
Logistic regression analysis of association between muscle composition phenotypes and low functional performance. (A) Self-reported slow walking pace; (B) Low handgrip strength; (C) Self-reported no stair climbing; and (D) Self-reported occurrence of more than one fall the last year. MFI, muscle fat infiltration.

Poor function was more common among participants with abnormal muscle composition (only low muscle volume, only high MFI, or AMC) compared with those with normal muscle composition (Fig 4; Table 4). CKD-participants with AMC had a significantly higher prevalence of low hand grip strength and slow walking pace (all *P* < 0.001) compared with those with normal muscle composition, only low muscle volume, or only high MFI (Fig 5; Table S1).

**Figure 6:**
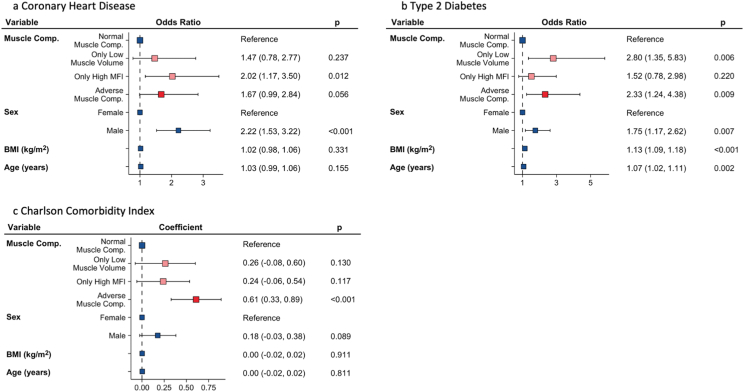
Logistic and linear regression analysis of association between muscle composition phenotypes and comorbidity. (A) Prevalent coronary heart disease; (B) Prevalent diabetes type 2; and (C) Charlson Comorbidity Index. MFI, muscle fat infiltration.

The CCI was higher in participants with abnormal muscle composition as compared with those with normal muscle composition, being significantly higher in the AMC-group (*P* < 0.001). The T2D prevalence was significantly higher in the AMC-group (*P* = 0.009) and in the only low muscle volume group (*P* = 0.006). The prevalence of CHD was significantly higher in participants with only high MFI (*P* = 0.01) (Fig 6; Table S1).

**Figure 7:**
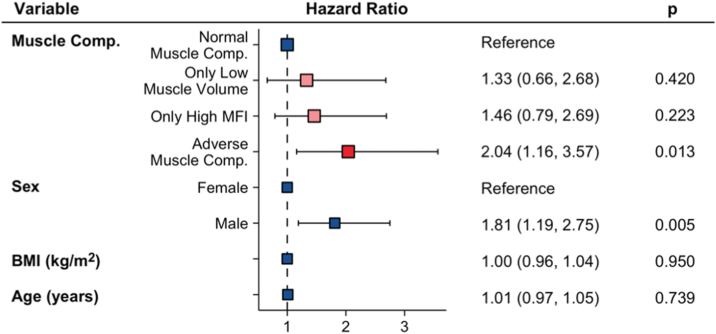
Cox proportional-hazard ratios of association between muscle composition phenotypes and incidence of coronary heart disease. MFI, muscle fat infiltration.

Of the 903 CKD participants, 100 individuals had a new CHD event during a mean follow-up time of 3.6 years (missing n = 5). The CHD incidence was 2 times higher in the AMC-group (*P* = 0.013) while the difference for the only high MFI or only low muscle volume did not show statistical significance (Fig 7).

## Discussion

In this study we show that MRI-based assessment of muscle composition is technically feasible and clinically relevant in CKD. The AMC is linked to comorbidity and poor physical function in individuals with NAFLD^9^ and this study extends the association to CKD. The AMC was prevalent both among dialysis patients in Sweden and in participants of the UK-Biobank study with CKD. The clinical relevance of AMC was assessed among dialysis patients with the correlation to comorbidity index and for CKD UK Biobank participants with correlation to muscle strength, comorbidity, and cardiovascular events during follow-up.

It has previously been reported that CKD-individuals have an increased ectopic fat deposition in skeletal muscle (MFI) with loss of muscle strength and function, which is often greater than the loss in skeletal muscle volume.^1^^,^^22^ Muscle fat infiltration is associated with poor clinical outcomes in CKD.^23^^,^^24^ Thus, in addition to muscle volume, the evaluation of MFI as a biomarker of muscle quality has an important role when assessing muscle composition. The reported prevalence of sarcopenia in CKD varies widely depending on the methods and cut-off criteria used to evaluate muscle mass. In fact, there is no consensus on which is the best skeletal muscle mass indicex for evaluating the adequacy of muscle mass^7^ and common ways to adjust muscle mass for body size (division by height^2^, weight, or BMI) do not effectively normalize this relationship.^8^ Eg, the sarcopenia prevalence according to EWGSOP2 was only around 3% in the CKD-UK Biobank group, whereas the prevalence of AMC was 32%. This is to some degree explained by the inability of the EWGSOP2 definition to correct the muscle volume for body size in the presence of overweight. In contrast, the muscle volume z-score used in the AMC definition is invariant to BMI.^8^ The AMC was a highly prevalent muscle composition phenotype in both the HD-and CKD-UK Biobank cohorts. One can speculate that the prevalence of AMC within CKD in clinical practice might be higher than estimated in this study, since the CKD prevalence in our study population was lower compared to general population or in UK Biobank studies.^25^^,^^26^ This is probably due to the combination of known healthy responder selection bias and younger age in the UK Biobank imaging study. The majority of CKD-UK Biobank participants in our study were in CKD stage 3 and the high prevalence of AMC in this relatively early CKD stage highlights the importance of the evaluation of muscle health in early disease states, as it often is overlooked.

CKD-UK Biobank participants with AMC were older and had a lower eGFR level than those without AMC. Chronic kidney disease associated sarcopenia is more common in older patients and associates with eGFR decline^27^ being more common in end-stage disease.^28^^,^^29^ The AMC has been associated with metabolic comorbidities in NAFLD.^9^ Conditions that are directly related to CKD pathogenesis, such as cardiovascular disease and diabetes mellitus, are also independently associated with sarcopenia.^30^^,^^31^ Our results showed that muscle composition was associated to the comorbidity burden as the Muscle_comb_ in the HD-cohort and AMC in the CKD-UK Biobank cohort were significantly associated with the comorbidity index. Indeed, AMC was associated to T2D in CKD-UK Biobank cohort. Furthermore, our study elucidates that muscle composition varies between CKD-individuals. Multiple factors are probably responsible for these differences but a higher T2D and CHD prevalence in individuals with abnormal muscle composition indicate that those phenotypes are associated with metabolic dysfunction and might constitute a group of interest for preventive interventions.

Muscle fat infiltration is recognized as a major risk factor for cardiovascular disease in general population and associates to cardiovascular events and mortality in dialysis patients.^23^^,^^24^^,^^32^ Recent findings suggest that in addition to MFI, the skeletal muscle mass has to be evaluated to predict cardiovascular events as preserved muscle mass may contribute to cardiovascular health in general population^33^ and low skeletal mass is associated with major adverse cardiovascular events in CKD.^34^ Therefore, it seems logical to evaluate both muscle composition biomarkers simultaneously when assessing the cardiovascular incidence in CKD, as we have done in our study. We found that AMC significantly increased the risk of CHD while the association with only low muscle volume and only high MFI was not statistically significant in this study. Thus, CKD patients with AMC seem to be at greater risk of CHD-outcomes. Considering that VAT reflects an excess of ectopic fat mass and is considered a major risk factor for metabolic and cardiovascular diseases in CKD,^35^^,^^36^ it is important to note that participants with AMC presented significantly higher VAT levels.

Sarcopenia associates with a decline in physical abilities and increase in disability.^37^ In our study, only AMC showed a significant association, which is consistent with Linge et al^12^ in NAFLD. This is also in line with another study assessing muscle composition with ultrasound in CKD patients that reported that both muscle size and muscle quality (MFI) may contribute to deficits in mobility and function.^38^

The MRI technique used in this study can aid in identifying patients at high risk of functional decline and adverse outcome. It could also contribute to the development of more effective, individualized clinical interventions, such as nutritional support, physical rehabilitation, or medical management, potentially improving prognosis and quality of life.

The strengths of this study, in addition to the prospective design and large sample size, are the use of cystatin C (muscle mass and function independent) for identifying CKD, the detailed assessment of muscle composition (muscle volume and MFI) with MRI, and the use of quantitative muscle biomarkers. The latter has strong associations to functional performance and comorbidity, thus providing a clinically meaningful description of muscle composition heterogeneity within CKD. In a similar fashion MRI-assessed muscle composition has recently been shown to be able, in contrast to other methods, to differentiate between adaptive and maladaptive lean tissue loss in pharmacologically induced weight loss.^39^ Additionally, the quantitative muscle biomarkers have also been validated across various MR scanner field strengths and manufacturers. For instance, Borga et al^40^ reported that the within-scanner repeatability coefficient was for 0.17 L for thigh muscle volume and 0.53 p.p. for muscle mass infiltration.

A limitation of the study is that blood samples defining CKD were taken 7-9 years before the MRI in this study. Given that the eGFR decline in large CKD cohorts has been reported to be 1.7-2 mL/min/year,^41^^,^^42^ the eGFR on the day of MRI is lower than reported in Table 2. This means that while some participants have progressed to a higher CKD stage, the majority—who were in stage 3a at baseline—would still be expected to remain within CKD stage 3 at the time of MRI. Although individual misclassification is possible, the overall CKD stage distribution at the time of MRI likely remained largely unchanged. More importantly, it does not affect any of the main conclusions of this study, such as the high prevalence of AMC, its association with comorbidities, and its prognostic relevance. Other limitations include that data regarding walking pace, number of falls, and stair climbing were self-reported; and T2D cases were identified through interviews, making the reliability of the data somewhat low. The CHD events, on the contrary, were identified through electronic health records.

## Conclusion

This study demonstrates that the novel MRI method, combining body size-adjusted muscle volume and MFI, can be utilized to analyze muscle composition in CKD patients. Further, its clinical relevance was demonstrated through associations with comorbidity, poor functional performance, and increased CHD risk. Our findings suggest that CKD patients with poor muscle health are a highly vulnerable group, and this technique may enable them to be targeted for accurate interventions in order to improve their outcomes.

## Supplementary Materials

Supplementary File (PDF) Item S1; Table S1.
