# Supplementary material for: Adverse Muscle Composition Is an Early Feature of Chronic Kidney Disease and Associates With Poor Function and Comorbidities
Source: Kidney Med. 2025 Oct 30;8(1):101164. doi: 10.1016/j.xkme.2025.101164 (PMC12768902; doi:10.1016/j.xkme.2025.101164)
Supplement: Supplementary File (PDF) — Item S1; Table S1. [file mmc1.pdf]

Item S1: Assessment of walking pace, number of falls and stair climbing were assessed through self-reported touchscreen questionnaires.

Walking pace, “Usual walking pace” (UK Biobank Field ID 924)
Screenshot from touchscreen questionnaire :

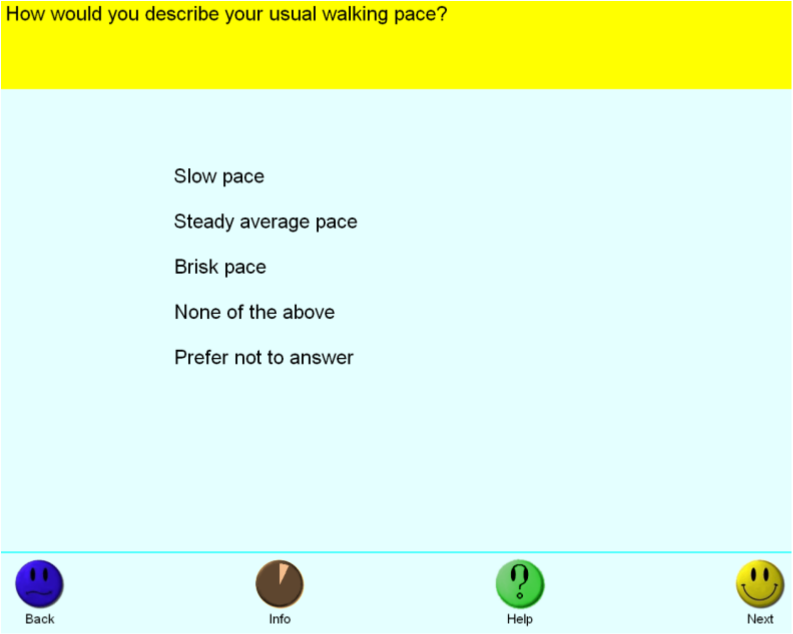

Falls, “Falls in the last year” (UK Biobank Field ID 2296)
Screenshot from touchscreen questionnaire :

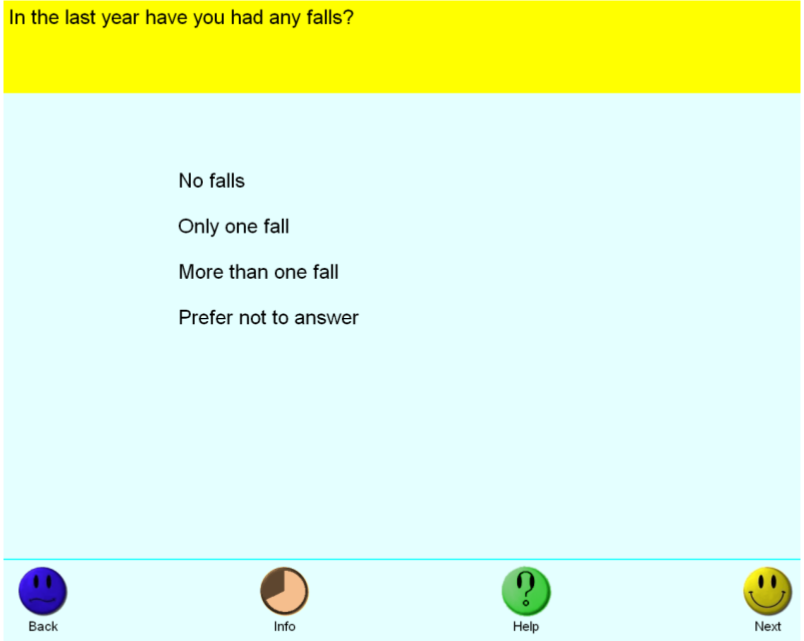

Stair climbing, “Frequency of stair climbing in last weeks” (UK Biobank Field ID 943)
Screenshot from touchscreen questionnaire:

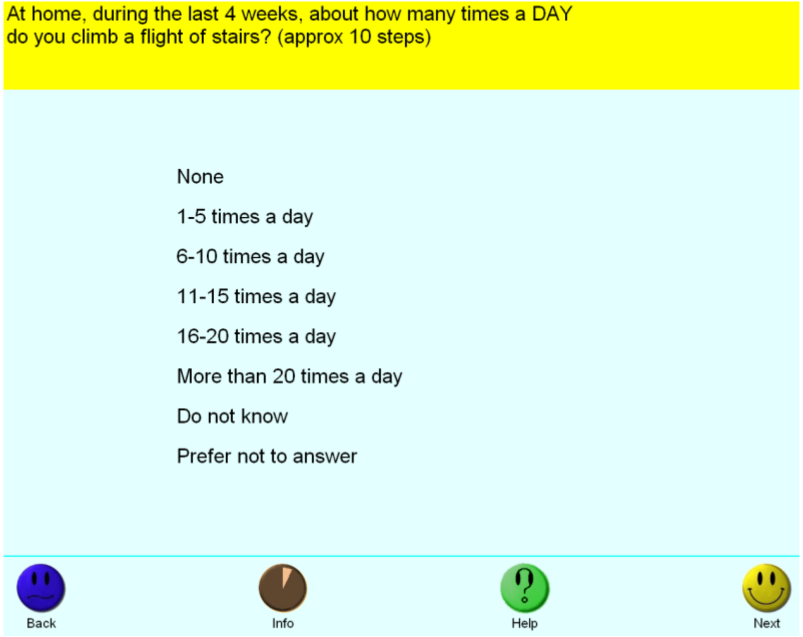
At home, during the last 4 weeks, about how many times a DAY do you climb a flight of stairs? (approx 10 steps)

None

1-5 times a day

6-10 times a day

11-15 times a day

16-20 times a day

More than 20 times a day

Do not know

Prefer not to answer

Back Info Help Next

Table S1. Comparison of the four different muscle composition phenotypes within the CKD-UK Biobank participants.

	Controls	CKD and normal muscle composition	CKD and only low muscle volume	CKD and only high muscle fat infiltration	CKD and adverse muscle composition
N	3612	245	123	242	293
Sex [female/male (%)]	47.8% / 52.2%	46.9% / 53.1%	40.7% / 59.3%	55.4% / 44.6%	45.4% / 54.6%
Age [years]	72.2 ± 5.8	70.2 ± 6.5	72.7 ± 5.6	72.0 ± 5.5	73.9 ± 5.1
Age at visit 1 [years]	61.7 ± 5.7	61.0 ± 6.3	62.9 ± 5.1	62.5 ± 5.3	63.6 ± 4.6
Height [m]	168.6 ± 9.3	169.9 ± 8.4	170.1 ± 9.4	168.2 ± 9.5	168.0 ± 8.9
Weight [kg]	82.4 ± 16.8	78.1 ± 14.0	75.1 ± 15.1	89.6 ± 18.7	84.2 ± 16.0
BMI [kg/m2]	29.0 ± 5.3	27.0 ± 4.2	25.9 ± 4.4	31.6 ± 5.6	29.8 ± 4.9
Kidney Function at Visit 1					
Cystatin C [mg/L]	0.9 ± 0.1	1.3 ± 0.2	1.3 ± 0.3	1.3 ± 0.1	1.3 ± 0.2
eGFR [mL/min/1.73m2]	84.9 ± 13.0	54.0 ± 6.4	53.7 ± 6.7	53.8 ± 5.5	52.8 ± 7.0
eGFR >60 [n (%)]	3612 (100.0%)	0 (0.0%)	0 (0.0%)	0 (0.0%)	0 (0.0%)
eGFR 59-45 [n (%)]	0 (0.0%)	222 (90.6%)	116 (94.3%)	225 (93.0%)	251 (85.7%)
eGFR 44-30 [n (%)]	0 (0.0%)	21 (8.6%)	6 (4.9%)	15 (6.2%)	38 (13.0%)
eGFR 29-15 [n (%)]	0 (0.0%)	2 (0.8%)	0 (0.0%)	2 (0.8%)	4 (1.4%)
eGFR <15 [n (%)]	0 (0.0%)	0 (0.0%)	1 (0.8%)	0 (0.0%)	0 (0.0%)
Lab Data at Visit 1					
Haemoglobin [g/dL]	14.3 ± 1.1	14.4 ± 1.4	14.4 ± 1.3	14.2 ± 1.4	14.3 ± 1.4
Albumin [g/L]	44.9 ± 2.5	44.3 ± 2.9	45.0 ± 2.5	44.5 ± 2.6	44.5 ± 2.8
HbA1c [mmol/mol]	36.4 ± 5.7	36.0 ± 4.6	37.4 ± 6.3	37.6 ± 5.4	38.3 ± 8.5
CRP [mg/L]	2.6 ± 4.3	3.5 ± 4.7	4.9 ± 9.3	4.5 ± 6.6	4.3 ± 4.7
Testosterone [nmol/L]	7.1 ± 5.9	7.7 ± 6.2	8.3 ± 5.9	6.2 ± 5.6	7.0 ± 5.5
uACR > 3 mg/mmol [%]	272 (7.7%)	35 (14.6%)	22 (18.2%)	25 (10.4%)	43 (14.9%)
Fat Distribution					
Visceral adipose tissue [L]	4.9 ± 2.6	4.2 ± 2.3	4.4 ± 2.0	5.7 ± 2.5	5.9 ± 2.6
Abdominal subcutaneous adipose tissue [L]	8.2 ± 3.8	6.8 ± 3.1	7.1 ± 3.5	9.9 ± 4.2	9.2 ± 3.9
Muscle Composition					
FFMV [L]	10.2 ± 2.4	10.7 ± 2.3	9.2 ± 2.0	10.7 ± 2.4	9.1 ± 2.0
Muscle volume z-score [SD]	-0.4 ± 0.9	0.3 ± 0.7	-1.2 ± 0.4	0.0 ± 0.6	-1.5 ± 0.6
MFI [%]	8.5 ± 2.2	6.9 ± 1.1	7.1 ± 1.0	10.3 ± 1.7	10.8 ± 2.4
MFI, sex adjusted [p.p.]	1.5 ± 2.1	-0.1 ± 0.9	0.2 ± 0.9	3.2 ± 1.7	3.8 ± 2.3
Adverse muscle composition [n (%)]	897 (24.8%)	0 (0.0%)	0 (0.0%)	0 (0.0%)	293 (100.0%)
Only high MFI [n (%)]	902 (25.0%)	0 (0.0%)	0 (0.0%)	242 (100.0%)	0 (0.0%)
Only low muscle volume [n (%)]	429 (11.9%)	0 (0.0%)	123 (100.0%)	0 (0.0%)	0 (0.0%)
Normal muscle composition [n (%)]	1384 (38.3%)	245 (100.0%)	0 (0.0%)	0 (0.0%)	0 (0.0%)
Functional Performance					
EWGSOP2 Sarcopenia [n (%)]	33 (1.5%)	1 (0.6%)	4 (4.5%)	0 (0.0%)	13 (6.8%)
Low hand grip strength [n (%)]	514 (14.7%)	26 (11.0%)	24 (20.3%)	36 (15.6%)	72 (25.4%)
No stair climbing [n (%)]	463 (13.0%)	30 (12.6%)	19 (16.1%)	20 (8.4%)	49 (17.4%)
Slow walking pace [n (%)]	433 (12.1%)	19 (7.9%)	14 (11.6%)	51 (21.3%)	77 (27.2%)
>1 fall last year [n (%)]	205 (5.7%)	15 (6.3%)	10 (8.3%)	20 (8.4%)	21 (7.2%)
Comorbidity					
Prevalent type 2 diabetes [n (%)]	313 (8.7%)	15 (6.1%)	19 (15.4%)	37 (15.4%)	59 (20.3%)
Prevalent coronary heart disease [n (%)]	340 (9.5%)	27 (11.1%)	20 (16.4%)	50 (20.7%)	56 (19.2%)
Charlson comorbidity index	0.5 ± 1.0	0.8 ± 1.4	1.1 ± 1.4	1.0 ± 1.5	1.4 ± 1.8

For continuous variables, data is reported as mean ± standard deviation. FFMV: fat free muscle volume, MFI: muscle fat infiltration, EWGSOP2: European Working Group on Sarcopenia in Older People.
